# Validity and diagnostics of the Italian version of the Montreal Cognitive Assessment (MoCA) in non-demented Parkinson’s disease patients

**DOI:** 10.1007/s40520-023-02493-w

**Published:** 2023-07-22

**Authors:** Alfonsina D’Iorio, Edoardo Nicolò Aiello, Marianna Amboni, Carmine Vitale, Federico Verde, Vincenzo Silani, Nicola Ticozzi, Andrea Ciammola, Barbara Poletti, Gabriella Santangelo

**Affiliations:** 1 Department of Psychology, University of Campania “Luigi Vanvitelli”, Caserta, Italy; 2 Department of Neurology and Laboratory of Neuroscience, IRCCS Istituto Auxologico Italiano, Milan, Italy; 3 Institute of Diagnosis and Health, IDC-Hermitage Capodimonte, Naples, Italy; 4 Department of Medicine, Surgery and Dentistry, University of Salerno, Salerno, Italy; 5 Department of Motor Sciences and Wellness, University “Parthenope”, Naples, Italy; 6 Department of Pathophysiology and Transplantation, “Dino Ferrari” Center, Università degli Studi di Milano, Milan, Italy; 7 Department of Oncology and Hemato-Oncology, Università degli Studi di Milano, Milan, Italy

**Keywords:** Montreal Cognitive Assessment, Parkinson’s disease, Cognitive screening, Neuropsychology

## Abstract

**Background:**

This study aimed at: (1) assessing, in an Italian cohort of non-demented Parkinson’s disease (PD) patients, the construct validity of the Montreal Cognitive Assessment (MoCA) against both first- and second-level cognitive measures; (2) delivering an exhaustive and updated evaluation of its diagnostic properties.

**Methods:**

A retrospective cohort of *N* = 237 non-demented PD patients having been administered the MoCA was addressed, of whom *N* = 169 further underwent the Mini-Mental State Examination (MMSE) and *N* = 68 the Parkinson’s Disease Cognitive Rating Scale (PD-CRS). A subsample (*N* = 60) also underwent a second-level cognitive battery encompassing measures of attention/executive functioning, language, memory, praxis and visuo-spatial abilities. Construct validity was assessed against both the PD-CRS and the second-level cognitive battery. Diagnostics were tested via receiver-operating characteristics analyses against a below-cut-off MMSE score.

**Results:**

The MoCA was associated with both PD-CRS scores (*p* < .001) and the vast majority of second-level cognitive measures (*p*s < .003). Both raw and adjusted MoCA scores proved to be highly accurate to the aim of identifying patients with MMSE-confirmed cognitive dysfunctions. A MoCA score adjusted for age and education according to the most recent normative dataset and < 19.015 is herewith suggested as indexing cognitive impairment in this population (AUC = .92; sensitivity = .92; specificity = .80).

**Discussion:**

The Italian MoCA is a valid and diagnostically sound screener for global cognitive inefficiency in non-demented PD patients. Further studies are nevertheless needed that confirm its diagnostic values against a measure other than the MMSE.

**Supplementary Information:**

The online version contains supplementary material available at 10.1007/s40520-023-02493-w.

## Background

Up to 40% of non-demented patients with Parkinson’s disease (PD) present with cognitive impairment [1] within both non-instrumental functions—*i.e.* attention and executive functioning—and instrumental domains—*i.e.* memory, visuo-spatial skills and language [2]. Since such dysfunctions detrimentally impact on patients’ functional outcomes [3] and prognosis [4], to screen for them via clinimetrically sound and feasible performance-based tests is clinically pivotal [1, 2] and thus highly advisable [5].

To this aim, according to the 2018 Movement Disorders Society (MDS) guidelines [5], the Montreal Cognitive Assessment (MoCA) [6] is—amongst those tests that are disease-nonspecific—strongly recommended. Such a screener has indeed received major support for use in this population by the International literature as far as its psychometrics, diagnostics as well as both cross-sectional and longitudinal feasibility are concerned [7], being also recommended within clinical trials as an outcome measure [8]. In fact, the MoCA samples from all of the abovementioned cognitive functions and domains are typically involved in PD [5–7].

However, with specific regard to the Italian scenario, the only two studies that focussed on the clinimetrics MoCA in non-demented PD patients—the first by Biundo et al*.* [9] and the second by Federico et al*.* [10]—are both lacking in relevant information and outdated.

Indeed, first, neither of these reports [9, 10] delivers evidence on the construct validity of the MoCA in this population—as both merely focussing on its diagnostic properties.

Moreover, although both Biundo et al*.* [9] and Federico et al*.* [10] overall provided data supporting the diagnostic value of the MoCA in non-demented PD patients, such studies either preceded the availability of demographically adjusted norms for the Italian MoCA (which were first delivered in 2015 [10,11])—as is the case for Biundo et al*.*’s [9] report—or addressed a limited sample size [6]—as is the case for Federico et al*.*’s [10] investigation, which included *N* = 43 patients. Moreover, Federico et al*.*’s [9, 10]  study, which dates back to 2015, of course could not address the most recent normative dataset for the MoCA—which has been updated in 2021 [12]. Taken together, such stances imply that, in Italy, no up-to-date, generalizable PD-specific cut-off is available for the MoCA [13].

Given the relevance of delivering comprehensive and up-to-date clinimetric information for a given cognitive screener, to the aim of increasing their level of recommendation for clinical and research use [14], the present study aimed at: (1) assessing, in an Italian cohort of non-demented PD patients, the construct validity of the MoCA against both first- and second-level cognitive measures; (2) delivering an exhaustive and updated evaluation of its diagnostic properties.

## Methods

### Participants

Data on *N* = 237 PD patients—diagnosed according to the UK Brain Bank criteria [15]—consecutively referred to IDC-Hermitage Capodimonte, Napoli, Italy between 2013 and 2019 and having undergone the MoCA were retrospectively collected. Patients did not present with dementia according to the DSM-5 criteria for a Major Neurocognitive Disorder due to PD [16]. Moreover, patients were free from (1) further neurological/psychiatric diseases, (2) severe and/or unstable general-medical conditions that could have possibly affected cognition (*i.e.* system/organ failures and uncompensated metabolic/internal conditions) 3) uncorrected hearing/vision deficits.

### Materials

**Fig. 1:**
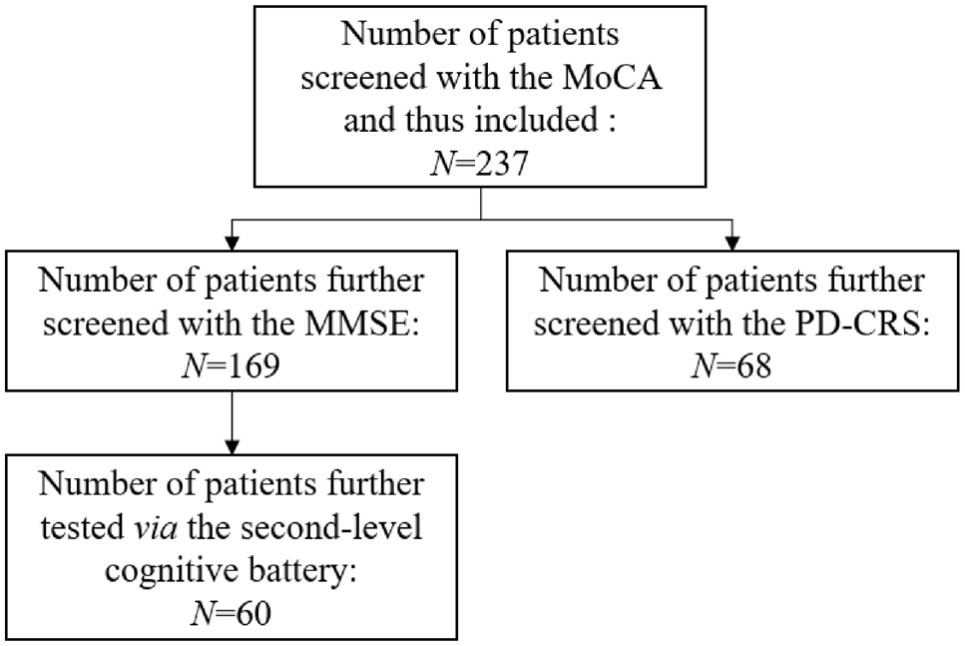
Flowchart displaying the tests administered to each patient subsample. *MoCA* Montreal Cognitive Assessment, *MMSE* Mini-Mental State Examination, *PD-CRS* Parkinson’s Disease Cognitive Rating Scale

All patients were administered the MoCA [13]. Patients assessed up to 2016 (*N* = 169) further underwent the Mini-Mental State Examination (MMSE) [17], whilst those assessed since 2017 (*N* = 68) were screened via the Parkinson’s Disease Cognitive Rating Scale (PD-CRS) [18]—the latter being is a 9-test, performance-based cognitive screening battery assessing attention/executive functioning, memory, language and visuo-spatial abilities/constructional praxis (*range* = 0–134). Out of patients assessed up to 2016 (*N* = 169), a subsample (*N* = 60) also underwent a second-level cognitive battery (Table 1) encompassing measures of attention and executive functioning (Trail-Making Test; Stroop Colour-Word Test; Phonemic Verbal Fluency; Backward Digit Span), memory (Rey Auditory Verbal Learning Test; Babcock Memory Test), language (Noun- and Verb-naming tasks from the Esame Neuropsicologico per l’Afasia; Semantic Verbal Fluency), praxis (Design Copy) and visuo-spatial abilities (Benton Judgment of Line Orientation) [19–25]. Figure 1 displays the abovementioned patient subsamples, defined according to which test they were administered.

**Table 1:** Second-level cognitive battery

Tests	Normative reference	Main target construct(s)
*Attention and executive functioning*		
Backward Digit Span	Monaco et al*.* [19]	Phonological working memory
SCWT-Word-naming	Barbarotto et al*.* [20]	Processing speed
SCWT-Colour-naming	Barbarotto et al*.* [20]	Processing speed
SCWT-Interference	Barbarotto et al*.* [20]	Inhibitory control
Trail-Making Test-A	Giovagnoli et al*.* [21]	Processing speed/selective attention
Trail-Making Test-B	Giovagnoli et al*.* [21]	Processing speed/dual attention
Trail-Making Test-B-A	Giovagnoli et al*.* [21]	Set-shifting abilities
Phonemic Verbal Fluency	Carlesimo et al*.* [22]	Inhibitory control
*Language*		
ENPA-Noun-naming	Capasso and Micieli [23]	Lexical retrieval/semantics
ENPA-Verb-naming	Capasso and Micieli [23]	Lexical retrieval/semantics
Semantic Verbal Fluency	Carlesimo et al*.* [22]	Lexical retrieval/semantics
*Memory*		
Rey Auditory Verbal Learning Test	Carlesimo et al*.* [22]	Episodic long-term memory
Babcock Memory Test	Spinnler & Tognoni [24]	Episodic long-term memory
*Visuo-spatial and praxic skills*		
Design Copy	Spinnler & Tognoni [24]	Constructional praxis
Benton Judgment of Line Orientation	Benton et al. [25]	Perceptual abilities

*SCWT* Stroop Colour-Word Test, *ENPA* Esame NeuroPsicologico per l’Afasia

### Statistics

Skewness and kurtosis statistics were addressed to check for Normality on raw variables (with values >|1| and |3| being judged as abnormal, respectively) [26]; thus, based on such an assumption being met or not, either parametric or non-parametric techniques were employed to test associations/predictions of interest. Skewness and kurtosis values for each cognitive measure are shown in Supplementary Table 1.

The construct validity of the MoCA against the PD-CRS was tested via a Pearson’s coefficient by addressing age- and education-adjusted scores on both tests [13, 18], whilst that against each measure of the second-level cognitive battery via a set of Bonferroni-corrected Spearman’s correlations that addressed test raw scores and covaried for age, education and sex.

The diagnostics of both raw and adjusted [13] MoCA scores were examined via receiver-operating characteristics (ROC) analyses by addressing a below-cut-off, age- and education-adjusted MMSE score [17] as the positive state. Such an operationalization was indeed supported both by a previous Italian report demonstrating the suitability of the MMSE for cognitive screening aims in this population [6], and by the fact that, within the present cohort, the effect size for the association between both raw (*r*_*s*_(169) = 0.64; *p* < 0.001) and adjusted scores (*r*_*s*_(169) = 0.53; *p* < 0.001) on the two tests was large—according to Cohen’s [27] benchmark (*i.e.*, ≥ 0.5). Sensitivity (Se), specificity (Sp), positive and negative predictive values (PPV; NPV) and likelihood ratios (LR + ; LR-) were computed at the optimal cut-off identified via Youden’s *J* statistic. The number needed for screening utility (NNSU) was also computed—with a value 1.02 ≤ being deemed as indexing an optimal screening performance [28].

Analyses were run with R 4.1 (R Core Team, 2012), jamovi 2.3 (the jamovi project, 2022) and IBM SPSS 27 (IBM Corp., 2020). The *Partial Correlation* macro of jamovi 2.3 was employed to compute partial Spearman’s coefficients.

## Results

Table 2 summarises patients’ background, clinical and cognitive measures.

**Table 2:** Patients’ background and cognitive measures

***N***	237
Sex (male/female)	168/69
Age (years)	66.4 ± 8.6 (34–88)
Education (years)	10.4 ± 4.6 (1–21)
Disease duration (years)	7.8 ± 4.1 (1–19)
UPDRS^*^	14.5 ± 6.7 (2–28)
H&Y (%)^*^	
Stage 1	7.8%
Stage 1.5	7.8%
Stage 2	42.2%
Stage 2.5	17.2%
Stage 3	20.3%
Stage 4	3.1%
LEDD^*^	759.1 ± 420.8 (100–2200)
MoCA (raw scores)	20.1 ± 4.6 (9–29)
Below-cut-off (%)	31.2%
MMSE (raw scores)^#^	26.8 ± 2.8 (14–30)
Below-cut-off (%)	14.8%
PD-CRS (raw scores)°	65.5 ± 20.2 (21–107)
Below-cut-off (%)	42.6%
Second-level cognitive battery^*§*^	
SCWT-Word-naming	50.2 ± 18 (12–89)
SCWT-Colour-naming	33.6 ± 11.2 (6–56)
SCWT-Interference	12.5 ± 7.4 (0–31)
Trail-Making Test-A	79.8 ± 65.8 (18–435)
Trail-Making Test -B	211.6 ± 152.1 (0–708)
Trail-Making Test -B-A	141.7 ± 108.2 (20–409)
Phonemic Verbal Fluency	26.3 ± 13.5 (7–67)
Backward Digit Span	3.3 ± 1.1 (1–6)
ENPA-Noun-naming	9.8 ± 0.4 (9–10)
ENPA-Verb-naming	9 ± 1 (6–10)
Semantic Verbal Fluency	16 ± 6 (4.8–33.8)
RAVLT-Immediate recall	33.2 ± 12.9 (11–64)
RAVLT-Delayed recall	6.4 ± 3.5 (0–15)
Babcock Memory Test	7.7 ± 4.6 (0–16)
Design Copy	11.2 ± 2.7 (4–14)
BJLO	16.6 ± 7.6 (0–30)

*PD* Parkinson’s disease, *UPDRS* Unified Parkinson’s Disease Rating Scales [29], *H&Y* Hoehn & Yahr scale [30] *LEDD* levodopa equivalent daily dose, *MoCA* Montreal Cognitive Assessment, *MMSE* Mini-Mental State Examination, *PD-CRS* Parkinson’s Disease Cognitive Rating Scale, *SCWT* Stroop Colour-Word Test, *RAVLT* Rey Auditory Verbal Learning Test, *BJLO* Benton Judgment of Line Orientation

^*^Data available for *N* = 64 patients;; ^#^data available for *N* = 169 patients; °data available for *N* = 68 patients; ^§^data available for *N* = 60 patients

The MoCA was significantly associated with both PD-CRS scores (*r*(68) = 0.68; *p* < 0.001) and to the vast majority of second-level cognitive measures (Table 3).

**Table 3:** Convergence of the MoCA against second-level cognitive measures

	MoCA
Attention and executive functioning	
SCWT-Word-naming	.50*
SCWT-Colour-naming	.52*
SCWT-Interference	.50*
Trail-Making Test-A	−.58*
Trail-Making Test -B	−.65*
Trail-Making Test -B-A	−.62*
Phonemic Verbal Fluency	.60*
Backward Digit Span	.28
Language	
ENPA-Noun-naming	.20
ENPA-Verb-naming	.54*
Semantic Verbal Fluency	.26
Memory	
Babcock Memory Test	.40
RAVLT-Immediate recall	.52*
RAVLT-Delayed recall	.40*
*Visuo-spatial abilities and praxis*	
Design Copy	.48*
BJLO	.55*

Spearman’s partial coefficients are displayed; age, education and sex were partialled out. *significant at α_adjusted_ = .003 *MoCA* Montreal Cognitive Assessment, *SCWT* Stroop Colour-Word Test, *RAVLT* Rey Auditory Verbal Learning Test, *BJLO* Benton Judgment of Line Orientation, *ENPA* Esame Neuropsicologico per L’Afasia

**Fig. 2:**
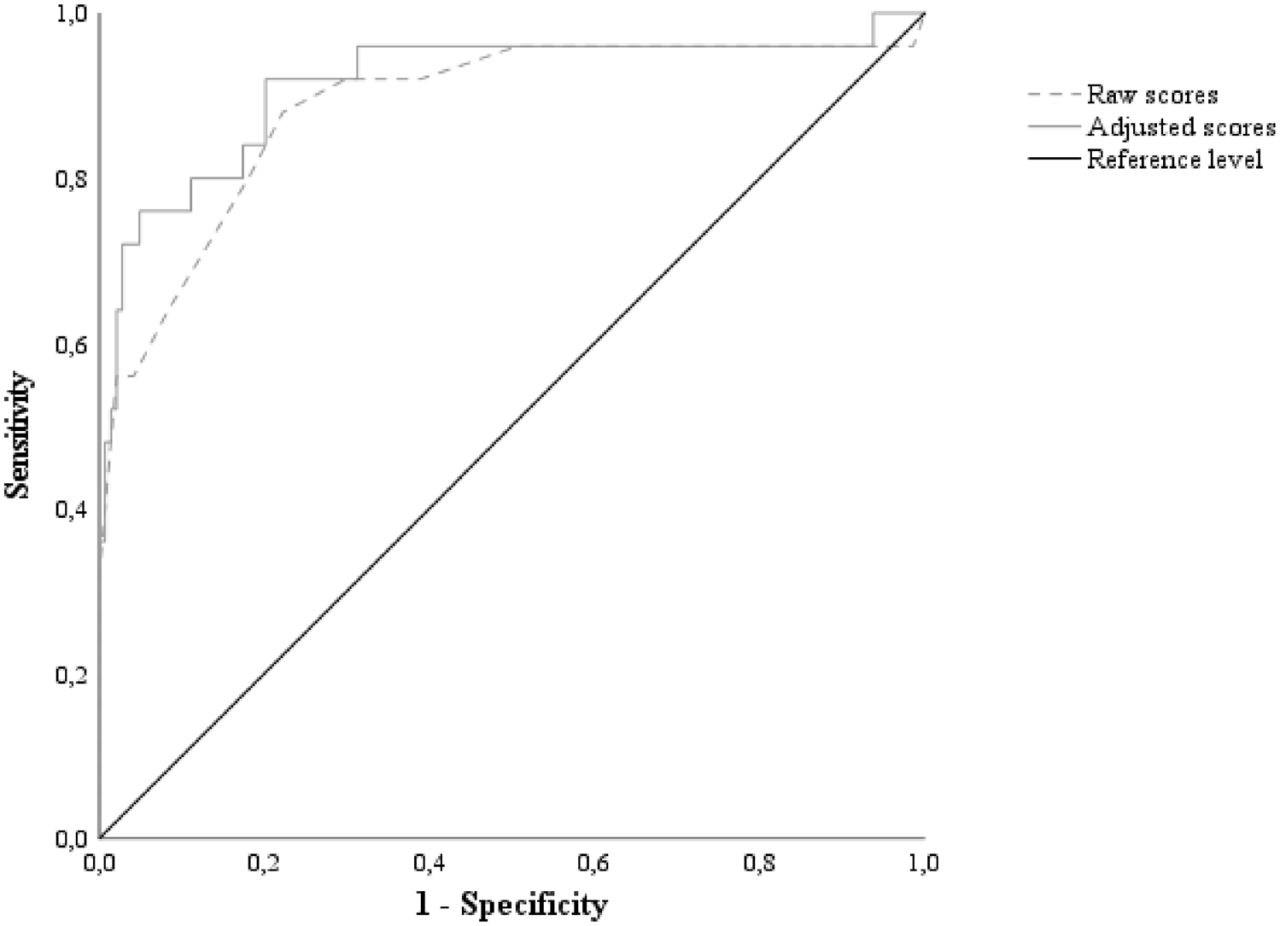
ROC curves for raw and adjusted MoCA against a defective MMSE score. **Notes.** ROC = receiver-operating characteristics; MoCA = Montreal Cognitive Assessment; MMSE = Mini-Mental State Examination. Raw scores (cut-off: ≤ 18): AUC = .89; *SE* = .04; CI 95% [.80, .97]; adjusted scores (cut-off: < 19.015): AUC = .91; *SE* = .04; CI 95% [.84, .99]. MoCA scores were adjusted according to Aiello et al*.* [9]

Twenty-five out of 169 patients performed defectively on the MMSE (14.8%). Both raw and adjusted MoCA scores proved to be highly accurate to the aim of identifying patients with MMSE-confirmed cognitive dysfunctions (Fig. 2), also coming with sound diagnostics at the optimal cut-offs of ≤ 18 (*J* = 0.66; Se = 0.88; Sp = 0.78; PPV = 0.41; NPV = 0.97; LR +  = 3.96; LR- = 0.15; NNSU = 0.89) and < 19.015 (*J* = 0.72; Se = 0.92; Sp = 0.80; PPV = 0.44; NPV = 0.98; LR +  = 4.57; LR- = 0.10; NNSU = 0.84), respectively.

When applied to the whole sample, the two cut-offs yielded two moderately consistent classifications—with their agreement rate being of 88% (Cohen’s *k* = 0.74; *z* = 11.38; *p* < 0.001). Discrepancies were accounted for by 14 patients being classified as impaired by the raw cut-off but not by the adjusted one and by other 14 patients classified as impaired by the adjusted cut-off but not by the raw one.

Notably, when exploratively testing the diagnostic properties of the optimal cut-off values previously derived on raw scores by Biundo et al*.* [9]—*i.e.*, ≤ 25—and by Federico et al*.* [10]—*i.e.*, ≤ 24— for detecting mild cognitive impairment in PD, accuracy was low (Biundo et al*.* [9]: 29%; Federico et al*.* [10]: 36%) and nominal metrics were excessively biassed towards Se as well as overall unacceptable (Biundo et al*.* [9]: Se = 0.96; Sp = 0.17; PPV = 0.17; NPV = 0.96; LR +  = 1.16; LR- = 0.23; NNSU = 3.06; Federico et al*.* [10]: Se = 0.96; Sp = 0.26; PPV = 0.18; NPV = 0.97; LR +  = 1.29; LR- = 0.16; NNSU = 2.35). Consistently, an unlikely extreme proportion of patients was classified as impaired according to such cut-offs (Biundo et al*.* [9]: 88%; Federico et al*.* [10]: 81%).

## Discussion

The present study provides Italian practitioners and clinical researchers with updated, and mostly unprecedented, evidence on the validity and diagnostic value of the MoCA in non-demented PD patients.

Indeed, the MoCA herewith proved to be an accurate estimate of cognitive efficiency in this population—as (1) being associated with a disease-specific measure of global cognition (*i.e.* the PD-CRS) and (2) converging with several second-level measures of both instrumental and non-instrumental cognitive domains/functions. Notably, such findings align with the international literature [5, 7].

As to its diagnostics, sound evidence has been herewith delivered on the fact that the MoCA is able to detect overall cognitive inefficiency (operationalized as a below-cut-off MMSE score) in non-demented PD patients. In this respect, it is worth noting that the cut-off derived on demographically adjusted MoCA scores slightly—but systematically—outperformed the “raw” cut-off. Such a finding is hardly surprising, given the undoubtable relevance of controlling for demographic confounders when interpreting test scores [14]. Hence, a MoCA score adjusted for age and education according to the most recent normative dataset [13] and falling below 19.015 should be addressed in the view of detecting cognitive impairment in this population.

In this respect, it is also worth noting that the raw cut-off herewith derived also clearly outperformed those previously suggested by Biundo et al*.* [9] and Federico et al*.* [10]—this strengthening, a posteriori*¸* the rationale underlying the present study (*i.e.* the need of delivering up-to-date and generalizable thresholds).

The present study is of course not free from limitations.

The first, and the most relevant, issue lies in the operationalization herewith adopted for the positive state—*i.e.* a below-cut-off MMSE score. Indeed, albeit evidence on its feasibility as a cognitive screener in PD patients has been delivered in Italy [10], it has to be borne in mind that the MMSE should not represent the preferred choice to the aim of screening for cognitive impairment in this population—as recently highlighted by the MDS [5]. Indeed, the MMSE is mostly unable to capture executive deficits—which, however, typically characterise the cognitive profile of PD [31, 32]. Hence, it is mandatory that future studies aim at replicating the present findings by employing a different operationalization of the positive state—*e.g.* by means of a second-level cognitive battery or via a disease-specific cognitive screener (such as the PD-CRS). With that being said, the strong association detected between MMSE and MoCA scores within the current cohort, as well as the sound diagnostics herewith yielded, suggests that a sufficient degree of generalizability might be granted to the present cut-offs.

As to the second, it should be noted that PPVs proved to be relatively poor—even more if addressed within the context of the remaining metrics. However, such a finding is not surprising and does not in any case undermine the overall optimal diagnostic performance of the MoCA herewith detected. Indeed, at variance with the LR + , PPVs are heavily influenced by the prevalence of the positive state in the study sample: the lower the prevalence (such is the case for this investigation), the lower the PPV.

A final limitation lies in fact that the normative dataset herewith addressed, by Aiello et al*.* [13], has been derived from individuals in Northern Italy—whilst the present patient cohort was recruited in Southern Italy. In this respect, Aiello et al*.* [13] themselves noted that, in healthy subjects, the three normative datasets that are currently available for the MoCA in Italy, which comes from individuals residing in different Italian regions, might classify individuals’ cognitive status differently [13]. However, it should be noted that Aiello et al*.*’s [13] normative dataset includes a larger number of individuals and covers a wider range of age and education when compared to the previous ones published by Conti et al*.* [11] and Santangelo et al*.* [12] between 2014 and 2015—this having led the present Authors to opt for Aiello et al*.*’s [13]. Whilst it would be far beyond the aim of this study to do so, future investigations might aim at exploring these three normative datasets classify PD patients differently as to their normative status—similarly to what Salvadori et al*.* [33] recently did in stroke patients. Finally, it is worth mentioning the present PD cohort was not particularly heterogeneous as far as their age and level of educational attainment were concerned; hence, the cut-offs herewith provided might not be representative of all ranges of age and education, and future investigations should be aimed at filling such a gap.

In conclusion, the Italian MoCA is a valid and diagnostically sound screener for global cognitive inefficiency in non-demented PD patients, whose adoption is recommended within both clinical and research settings. Further studies are nevertheless needed that confirm its diagnostic values against a measure that is more appropriate than the MMSE.

## Ethics

Participants provided informed consent. This study was approved by the Ethics Committee the University of Campania “Luigi Vanvitelli”.

### Supplementary Information

Below is the link to the electronic supplementary material.

Supplementary file1 (DOCX 15 KB)

## Data Availability

Datasets associated with the present study are available upon reasonable request of interested researchers.

## References

[1] Baiano C, Barone P, Trojano L, Santangelo G (2020). Prevalence and clinical aspects of mild cognitive impairment in Parkinson's disease: a meta-analysis. Mov Disord.

[2] Papagno C, Trojano L (2018). Cognitive and behavioral disorders in Parkinson’s disease: an update. I: cognitive impairments. Neurol Sci.

[3] Marras C, Rochon P, Lang AE (2002). Predicting motor decline and disability in Parkinson disease: a systematic review. Arch Neurol.

[4] Pedersen KF, Larsen JP, Tysnes OB, Alves G (2013). Prognosis of mild cognitive impairment in early Parkinson disease: the Norwegian ParkWest study. JAMA Neurol.

[5] Skorvanek M, Goldman JG, Jahanshahi M, Marras C, Rektorova I, Schmand B (2018). Global scales for cognitive screening in Parkinson's disease: Critique and recommendations. Mov Disord.

[6] Nasreddine ZS, Phillips NA, Bédirian V, Charbonneau S, Whitehead V, Collin I (2005). The Montreal Cognitive Assessment, MoCA: a brief screening tool for mild cognitive impairment. J Am Geriatr Soc.

[7] Julayanont P, Phillips N, Chertkow H, Nasreddine ZS (2017) The Montreal Cognitive Assessment (MoCA): Concept and Clinical Review. A.J. Larner (Ed.) Cognitive Screening Instruments: A Practical Approach (pp. 111–152). Springer

[8] Chou KL, Amick MM, Brandt J, Camicioli R, Frei K, Gitelman D (2010). A recommended scale for cognitive screening in clinical trials of Parkinson's disease. Mov Disord.

[9] Biundo R, Weis L, Facchini S, Formento-Dojot P, Vallelunga A, Pilleri M, Antonini A (2014). Cognitive profiling of Parkinson disease patients with mild cognitive impairment and dementia. Parkinsonism Relat Disord.

[10] Federico A, Maier A, Vianello G, Mapelli D, Trentin M, Zanette G (2015). Screening for mild cognitive impairment in Parkinson’s disease: comparison of the Italian versions of three neuropsychological tests. Parkinson’s Dis.

[11] Conti S, Bonazzi S, Laiacona M, Masina M, Coralli MV (2015). Montreal Cognitive Assessment (MoCA)-Italian version: regression based norms and equivalent scores. Neurol Sci.

[12] Santangelo G, Siciliano M, Pedone R, Vitale C, Falco F, Bisogno R (2015). Normative data for the Montreal Cognitive Assessment in an Italian population sample. Neurol Sci.

[13] Aiello EN, Gramegna C, Esposito A, Gazzaniga V, Zago S, Difonzo T (2022). The Montreal Cognitive Assessment (MoCA): updated norms and psychometric insights into adaptive testing from healthy individuals in Northern Italy. Aging Clin Exp Res.

[14] Aiello EN, Rimoldi S, Bolognini N, Appollonio I, Arcara G (2022). Psychometrics and diagnostics of Italian cognitive screening tests: a systematic review. Neurol Sci.

[15] Hughes AJ, Daniel SE, Kilford L, Lees AJ (1992). Accuracy of clinical diagnosis of idiopathic Parkinson's disease: a clinico-pathological study of 100 cases. J Neurol Neurosurg Psychiatry.

[16] American Psychiatric Association (2013) Diagnostic and statistical manual of mental disorders(5th ed.). American Psychiatric Association

[17] Carpinelli Mazzi M, Iavarone A, Russo G, Musella C, Milan G, D’Anna F (2020). Mini-Mental State Examination: new normative values on subjects in Southern Italy. Aging Clin Exp Res.

[18] Santangelo G, Lagravinese G, Battini V, Chiorri C, Siciliano M, Abbruzzese G (2017). The Parkinson’s disease-cognitive rating scale (PD-CRS): normative values from 268 healthy Italian individuals. Neurol Sci.

[19] Monaco M, Costa A, Caltagirone C, Carlesimo GA (2013). Forward and backward span for verbal and visuo-spatial data: standardization and normative data from an Italian adult population. Neurol Sci.

[20] Barbarotto R, Laiacona M, Frosio R, Vecchio M, Farinato A, Capitani E (1998). A normative study on visual reaction times and two Stroop colour-word tests. The Ital J Neurol Sci.

[21] Giovagnoli AR, Del Pesce M, Mascheroni S, Simoncelli M, Laiacona M, Capitani E (1996). Trail making test: normative values from 287 normal adult controls. Neurol Sci.

[22] Carlesimo, G. A., Caltagirone, C., Gainotti, G. & the MDB Group (1996). The Mental Deterioration Battery: Normative data, diagnostic reliability and qualitative analyses of cognitive impairment. Eur Neurol.

[23] Capasso R, Miceli G (2001) Esame Neuropsicologico per l'Afasia: ENPA. Springer Science & Business Media

[24] Spinnler H, Tognoni G (1987). Standardizzazione e taratura italiana di una batteria di test neuropsicologici. Neurol Sci.

[25] Benton AL, Varney NR, Hamsher KD (1978). Visuospatial judgment: A clinical test. Arch Neurol.

[26] Kim HY (2013). Statistical notes for clinical researchers: assessing normal distribution (2) using skewness and kurtosis. Restor Dent Endod.

[27] Cohen J (1988). Statistical power analysis for the behavioral sciences.

[28] Larner AJ (2019). New unitary metrics for dementia test accuracy studies. Prog Neurol Psychiatry.

[29] Fahn S, Elton RL (1987) UPDRS program members. Unified Parkinson’s disease rating scale. In: Fahn S, Marsden CD, Goldstein M, Calne DB (eds) Recent developments in Parkinson’s disease, pp 153–163

[30] Jankovic J, McDermott M, Carter J et al (1990). Variable expression of Parkinson's disease: a base-line analysis of the DAT ATOP cohort. Neurology.

[31] Hoops S, Nazem S, Siderowf AD, Duda JE, Xie SX, Stern MB, Weintraub D (2009). Validity of the MoCA and MMSE in the detection of MCI and dementia in Parkinson disease. Neurology.

[32] Chou KL, Lenhart A, Koeppe RA, Bohnen NI (2014). Abnormal MoCA and normal range MMSE scores in Parkinson disease without dementia: cognitive and neurochemical correlates. Parkinsonism Relat Disord.

[33] Salvadori E, Cova I, Mele F, Pomati S, Pantoni L (2022). Prediction of post-stroke cognitive impairment by Montreal Cognitive Assessment (MoCA) performances in acute stroke: comparison of three normative datasets. Aging Clin Exp Res.

