# Supplementary material for: Validity and diagnostics of the Italian version of the Montreal Cognitive Assessment (MoCA) in non-demented Parkinson’s disease patients
Source: Aging Clin Exp Res. 2023 Jul 22;35(10):2157–63. doi: 10.1007/s40520-023-02493-w (PMC10519859; doi:10.1007/s40520-023-02493-w)
Supplement: Supplementary file 1 — Supplementary file1 (DOCX 15 KB) [file 40520_2023_2493_MOESM1_ESM.docx]

**Supplementary table 1.** Skewness and kurtosis values of cognitive measures.

| **Measure** | **Skewness** | **Kurtosis** |
| --- | --- | --- |
| MMSE (raw scores) | -1.45 | 3.05 |
| MMSE (adjusted scores) | -1.38 | 3.17 |
| MoCA (raw scores) | -0.28 | -0.61 |
| MoCA (adjusted scores) | -0.30 | -0.18 |
| PD-CRS (raw scores) | -0.21 | -0.72 |
| PD-CRS (adjusted scores) | -0.01 | -0.93 |
| RAVLT-Immediate recall | 0.67 | -0.21 |
| RAVLT-Delayed recall | 0.57 | 0.23 |
| Design Copy | -1.14 | 0.73 |
| BJLO | -0.40 | -0.58 |
| ENPA-Noun-naming | -1.25 | -0.46 |
| ENPA-Verb-naming | -0.95 | 0.67 |
| Phonemic Verbal Fluency | 0.93 | 0.76 |
| Semantic Verbal Fluency | 0.48 | -0.07 |
| Babcock Memory Test | -0.04 | -0.70 |
| Backward Digit Span | 0.22 | 0.69 |
| SCWT-Word-naming | -0.04 | -0.61 |
| SCWT-Color-naming | 0.14 | -0.58 |
| SCWT-Interference | 0.42 | -0.55 |
| Trail-Making Test-A | 3.19 | 14.26 |
| Trail-Making Test -B | 1.15 | 0.87 |
| Trail-Making Test -B-A | 0.76 | -0.64 |

**Notes.** MMSE=Mini-Mental State Examination; MoCA=Montreal Cognitive Assessment; PD-CRS=Parkinson’s Disease Cognitive Rating Scale; SCWT=Stroop Color-Word Test; RAVLT=Rey Auditory Verbal Learning Test; BJLO=Benton Judgment of Line Orientation; ENPA=Esame Neuropsicologico per L’Afasia.
